# Differential rates of cesarean delivery by maternal geographical origin: a cohort study in France

**DOI:** 10.1186/s12884-019-2364-x

**Published:** 2019-06-27

**Authors:** Morgane Linard, Catherine Deneux-Tharaux, Dominique Luton, Thomas Schmitz, Laurent Mandelbrot, Candice Estellat, Priscille Sauvegrain, Elie Azria, for the PreCARE study group, Elie Azria, Béatrice Blondel, Agnès Bourgeois-Moine, Pierre François Ceccaldi, Catherine Crenn-Hebert, Guillaume Ducarme, Candice Estellat, Christelle Lecler, Dominique Luton, Jean-François Oury, Philippe Ravaud, Thomas Schmitz, Jeanne Sibiude, the BiP study group, Olivia Anselem, Elie Azria, Marie-Pierre Bonnet, Marguerite Cognet, Catherine Deneux-Tharaux, Sylvie Duquesnois, Romain Guedj, Morgane Linard, Charlotte Ngo, Juliette Richetin, Anne Rousseau, Marie-Josèphe Saurel, Priscille Sauvegrain

**Affiliations:** 1 INSERM U1153 – Obstetrical, Perinatal and Pediatric Epidemiology (EPOPé research team), DHU Risks in Pregnancy, Paris Descartes University, 53 Avenue de l’Observatoire, 75014 Paris, France; 2 Department of Obstetrics and Gynecology, Bichat Hospital, DHU Risks in Pregnancy, APHP, Paris Diderot University, Paris, France; 3 Department of Obstetrics and Gynecology, Robert Debré Hospital, AP-HP, Paris Diderot University, Paris, France; 4 Department of Obstetrics and Gynecology, Louis Mourier Hospital, DHU Risks in Pregnancy, APHP, Paris Diderot University, Colombes, France; 5 INSERM UMR 1123, CIC-P 1421, Department of Biostatistics, Public Health and Medical Information, Clinical research unit, Pharmacoepidemiology center (Céphépi), Pitié-Salpêtrière Hospital, APHP, Paris, France; 6 Department of Obstetrics, Paris Saint Joseph Hospital, DHU Risks in Pregnancy, Paris Descartes University, Paris, France

**Keywords:** Cesarean delivery, Differential care, Health disparities, Maternal geographical origin, Robson classification, Sub-Saharan Africa

## Abstract

**Background:**

In many Western countries, higher rates of cesarean have been described among migrant women compared to natives of receiving countries. We aimed to estimate this difference comparing women originating from France and Sub-Saharan Africa (SSA), identify the clinical situations explaining most of this difference and assess whether maternal origin was independently associated with cesarean risk.

**Methods:**

The PreCARE prospective multicenter cohort study was conducted in 2010–2012 in the north Paris area. Our sample was restricted to 1500 women originating from Sub-Saharan Africa and 2206 from France. Profiles of cesarean section by maternal origin were described by the Robson classification. Independent associations between maternal origin and 1) cesarean before labor versus trial of labor, then 2) intrapartum cesarean versus vaginal delivery were assessed by logistic regression models to adjust for other maternal and pregnancy characteristics.

**Results:**

Rates of cesarean for women originating from France and SSA were 17 and 31%. The Robson 5A category “unique uterine scar, single cephalic ≥37 weeks” was the main contributor to this difference. Within this category, SSA origin was associated with cesarean before labor after adjustment for medical risk factors (adjusted odds ratio [aOR] = 2.30 [1.12–4.71]) but no more significant when adjusting on social deprivation (aOR = 1.45 [0.63–3.31]). SSA origin was associated with cesarean during labor after adjustment for both medical and social factors (aOR = 2.95 [1.35–6.44]).

**Conclusions:**

The wide difference in cesarean rates between SSA and French native women is mainly explained by the Robson 5A category. Within this group, medical factors alone do not explain the increased risk of cesarean in SSA women.

**Electronic supplementary material:**

The online version of this article (10.1186/s12884-019-2364-x) contains supplementary material, which is available to authorized users.

## Background

Cesarean delivery is a life-saving procedure in some clinical situations but remains associated with increased risks for mothers and their babies as compared with vaginal delivery. Indeed, previous studies reported increased risks of maternal mortality and morbidity in the short term and in subsequent pregnancies as well as neonatal respiratory morbidity and adverse child health events after a cesarean delivery [1–10].

To avoid unnecessary interventions and risks for the mothers and their babies, health authorities recommend reducing the rates of cesarean by identifying situations with medically unjustified indications [11–14]. In this perspective, focusing on subgroups known to be at high risk of cesarean section would help to better understand the reasons for this and avoid unnecessary cesarean sections. Migrant women from non-Western countries, despite a wide heterogeneity of this category, appear to be at higher risk than those from host countries, especially women from Sub-Saharan Africa (SSA) [15–17]. However, whether this more frequent use of cesarean delivery is medically justified remains unclear. A higher prevalence of medical risk factors among those women could be an explanation and needs to be addressed [17, 18]. However, some authors suggest that nonmedical factors such as communication barriers, support during labor and birth, and more distant factors such as low socioeconomic status, may be important determinants of cesarean delivery in these populations [15, 19, 20]. Finally, as described in other health settings, implicit bias in care givers may contribute to differential care [21].

In a French cohort characterized by a large proportion of women from SSA, we aimed to quantify the difference in rates of cesarean between women from France and from SSA, identify the clinical category explaining most of this difference, and assess whether the differential use of cesarean delivery in SSA women in this clinical category is explained by medical factors.

## Methods

### Study design

The PreCARE prospective multicenter cohort study took place between 2010 and 2012 in four maternity units in university hospitals in northern Paris (France) [22]. This study, whose primary goal was to assess the association between social deprivation and maternal and perinatal morbidity, was conducted in a geographical area characterized by its high prevalence of social deprivation and a wide multiculturalism.

The regional review board approved the study (CPP-Ile-de-France III, no. 09.341bis, November 19, 2009). Each woman provided oral informed consent, in compliance with French law. This study was supported by grants from Medical Research Foundation (http://www.frm.org/), French Ministry of Health, PHRC 2007 and PHRC 2012 (https://solidarites-sante.gouv.fr/systeme-de-sante-et-medico-social/recherche-et-innovation/l-innovation-et-la-recherche-clinique/appels-a-projets/article/le-programme-hospitalier-de-recherche-clinique-phrc). The funders had no role in study design, data collection and analysis, decision to publish, or preparation of the manuscript.

### Participants

**Fig. 1:**
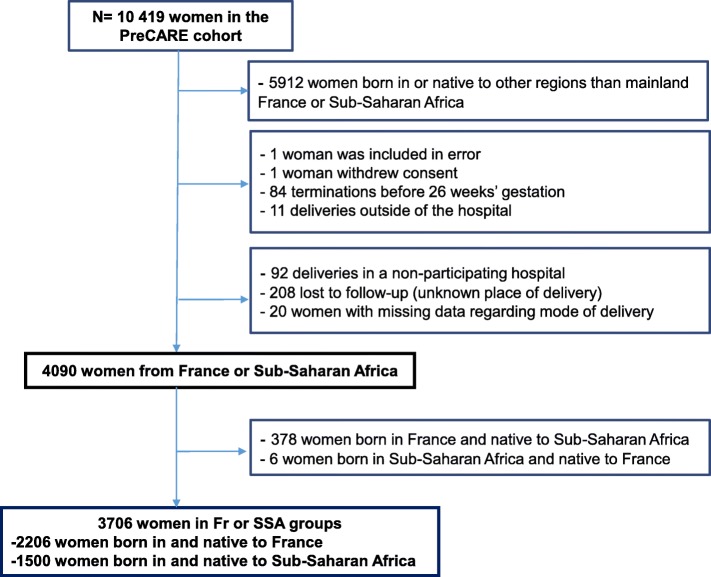
Flow of women in the study**.** Abbreviations: Fr = French, SSA = Sub-Saharan Africa

Participants in the PreCARE cohort were all women ≥18 years registered to deliver or who delivered in these units (*n* = 10,419). For this secondary analysis, we excluded women who were born in or were native to other regions than mainland France or Sub-Saharan Africa (*n* = 5912). We also excluded women who gave birth before 26 completed weeks’ gestation (*n* = 84) to avoid situations in which absence of active care of the newborn could affect the mode of delivery; and women who gave birth outside of the hospital (*n* = 11), women who gave birth in a non-participating hospital (*n* = 92), were lost to follow-up (*n* = 208) and for whom the mode of delivery was unknown (*n* = 20). Finally, our study sample comprised 4090 women from France or SSA. (Flow chart in Fig. 1).

### Available data

Sociodemographic data were collected by self-administered questionnaires (one at inclusion and one during the post-partum period before discharge). These questionnaires were available in French and English. In case of a linguistic barrier or difficulties in reading or writing, help was provided by a research assistant or an interpreter.

Women’s medical history and information about their pregnancy and delivery were collected by research assistants and practitioners by specific questionnaires completed in the immediate postpartum period and before maternity discharge.

The exposure of interest (i.e., maternal origin) was defined by the combination of the mother’s place of birth and the self-declared geographical origin. This combination led to the classification of women into four groups: 1) women born in mainland France and originally from mainland France (*n* = 2206), 2) women born in SSA and originally from SSA (*n* = 1500), 3) women born in mainland France and originally from SSA (*n* = 378) and 4) women born in SSA and originally from mainland France (*n* = 6). Because of the small size of the two last groups and to limit heterogeneity within each compared group, we restricted our study population to the first two groups (*n* = 3706) which we refer to as “Fr group” and “SSA group”.

Profiles of cesarean delivery among Fr and SSA groups were described and compared by the Robson classification [23], which is recommended by the World Health Organization and based on objective parameters that are easily reproducible and clinically pertinent [24]. This classification defines 10 exclusive categories of women based on six factors: parity, previous uterine scar (including previous cesarean section and/or uterine scar after gynecological surgery), number of fetuses, fetal presentation, gestational age at delivery and onset of labor. Categories 1 to 4 generally concern “women at low risk”: nulliparous or multiparous without uterine scars, singleton, cephalic, ≥37 weeks. Categories 5 to 10 concern “women at high risk”: history of uterine scar, breech and abnormal lies, multiple gestations, preterm delivery. Complete definitions of each category are available in Table 2.

Variables related to mother’s medical history (maternal age, body mass index, parity, history of uterine scar, medical risk level at the beginning of pregnancy), sociodemographic characteristics (education, social deprivation, linguistic barrier, legal status, length of stay in France) and characteristics of the pregnancy (number of fetuses, adequacy of prenatal care, complications during pregnancy, large or small for gestational age, preterm delivery [i.e., < 37 weeks’ gestation], fetal presentation, onset of labor, maternity unit of delivery) were used as covariates.

High medical risk level at the beginning of pregnancy, social deprivation and adequacy of prenatal care were defined as proposed in Linard et al. [22]. Complications during pregnancy was a binary variable defined as the occurrence of one or more of the following: gestational diabetes, diabetic ketoacidosis, pre-eclampsia, eclampsia, placenta abruption, HELLP syndrome, venous thrombosis, pulmonary embolism, severe sepsis, convulsions, coagulation disorder, cholestasis of pregnancy and complications due to uterine fibroids.

### Statistical analysis

The Fr and SSA groups were described, and profiles of cesarean delivery by maternal origin were described by using the Robson classification. For each category of the Robson classification, the relative size of the category (number of women in each category divided by the total number of women), the cesarean rate (number of cesarean deliveries in the category divided by the number of women in the category), and the absolute contribution to the total cesarean rate (number of cesarean deliveries in each category divided by the total number of women) were calculated and compared between the Fr and SSA groups to identify the clinical category explaining most of the difference in cesarean rates.

Within the Robson clinical category explaining most of the difference in cesarean rates, analyses were performed for 1) cesarean delivery before labor (vs trial of labor) and 2) intrapartum cesarean (vs vaginal delivery) among women in labor. Multivariate logistic regression models were created to assess the direct effect of maternal geographical origin on cesarean delivery after adjustment for both confounding and intermediate factors [25]. The variables introduced into the models were those clinically relevant or found in the literature [15, 16]. To distinguish the effect of medical risk factors of cesarean delivery from the one of social risk factors, we used two models: one adjusting for medical risks and the other adjusting for both medical risks and social deprivation. The assumptions of log-linearity and goodness-of-fit were verified. The missing data rates for the variables included in the models ranged from 0 to 7.6% (details in Additional file 1: Tables S1 and Additional file 2: Table S2). To retain women with missing data in the analyses, we performed multiple imputations by chained equations [26, 27]. Imputed data were women’s height and weight (to calculate the body mass index), medical risk level at the beginning of pregnancy, realization of ultrasound examinations (to determine the adequacy of prenatal care) and the estimation of fetal weight. The results of the models are presented as adjusted odds ratios (aORs) with their 95% confidence intervals (CIs).

A sensitivity analysis was performed using an alternative definition of maternal geographical origin defined by mother’s place of birth only, as recommended by the Reproductive Outcome And Migration (ROAM: an international collaboration) and EURO-PERISTAT [28].

To discuss a potential selection bias, we compared the characteristics of the women included in the analysis (*n* = 3706) and women from the Fr or SSA group excluded because they gave birth in a non-participating hospital, were lost to follow-up or had missing data concerning their mode of delivery (*n* = 301).

All statistical tests were two-tailed and the threshold for statistical significance was 5%. Analyses involved use of Stata v12.1 (Stata Corp., College Station, TX, USA).

## Results

Characteristics of women are in Table 1. SSA women had more potential risk factors of cesarean delivery than Fr women: overweight, obesity, high medical risk level at the beginning of pregnancy, previous uterine scar (particularly multiple uterine scars), inadequate antenatal care utilization, complications during pregnancy, social deprivation, and low level of education.

**Table 1:** Characteristics of women from France and Sub-Saharan African origin

Characteristic	Fr Group (*n* = 2206)		SSA Group (*n* = 1500)		P
	n	%	n	%	
Age (years)					
< 25	195	(8.8)	223	(14.9)	< 0.01
25–29	611	(27.7)	458	(30.5)	
30–34	891	(40.4)	455	(30.3)	
≥ 35	509	(23.1)	364	(24.3)	
Education					
≤ Primary school	5	(0.2)	329	(22.3)	< 0.01
Middle school	236	(10.7)	355	(24.0)	
High school	280	(12.7)	384	(26.0)	
University	1682	(76.4)	410	(27.7)	
Social deprivation^a^					
none	1978	(89.7)	589	(39.3)	< 0.01
1 criterion	137	(6.2)	358	(23.9)	
2 criteria	65	(2.9)	224	(15.0)	
3 or 4 criteria	26	(1.2)	326	(21.8)	
Body mass index (kg/m^2^)					
< 24.9	1697	(78.2)	649	(49.2)	< 0.01
25–29.9	286	(13.2)	396	(30.0)	
≥30	187	(8.6)	275	(20.8)	
Parity					
0	1239	(56.2)	412	(27.5)	< 0.01
1	680	(30.8)	418	(27.9)	
≥2	286	(13.0)	669	(44.6)	
High medical risk level at the beginning of pregnancy^b^	384	(17.5)	399	(26.7)	< 0.01
Previous uterine scar^c^					
0	2029	(92.0)	1159	(77.4)	< 0.01
1	147	(6.7)	230	(15.4)	
≥2	29	(1.3)	109	(7.3)	
Multiple pregnancy	77	(3.5)	60	(4.0)	0.42
Adequacy of prenatal care utilization^d^					
Inadequate	454	(21.4)	669	(46.5)	< 0.01
Intermediate	306	(14.4)	226	(15.7)	
Adequate	501	(23.6)	243	(16.9)	
Adequate plus	865	(40.7)	300	(20.9)	
Preterm delivery	207	(9.4)	150	(10.0)	0.52
Estimation of fetal weight^e^					
Small for gestational age	84	(4.0)	90	(6.4)	< 0.01
Normal	1988	(94.9)	1289	(91.5)	
Large for gestational age	23	(1.1)	29	(2.1)	
Complications of pregnancy^f^	241	(10.9)	213	(14.2)	< 0.01
Fetal presentation^g^					
Cephalic	2083	(94.7)	1424	(95.3)	< 0.01
Breech	113	(5.1)	59	(3.9)	
Transverse	3	(0.1)	12	(0.8)	
Onset of labor					
Spontaneous	1498	(67.9)	875	(58.3)	< 0.01
Induction	569	(25.8)	401	(26.7)	
Cesarean delivery before labor	139	(6.3)	224	(14.9)	
Mode of delivery					
Cesarean delivery	370	(16.8)	458	(30.5)	< 0.01

*Abbreviations*: *Fr group* women born in mainland France and originally from mainland France, *SSA group* women born in Sub-Saharan Africa and originally from Sub-Saharan Africa

^a^Defined as the presence of one or more of: 1) social isolation; 2) unstable or insecure housing conditions; 3) main household income not due to paid work; 4) lack of standard health insurance

^**b**^High medical risk level at the beginning of pregnancy was defined as the presence of one or more of: history of cardiac disease, hypertension, diabetes, venous thrombosis, pulmonary embolism, Graves’ disease, asthma, homozygous sickle cell anemia, thrombocytopenia, coagulation disorder, a rare or systemic disease, nephropathy, HIV infection, late miscarriage, pre-eclampsia, growth restriction, preterm delivery, fetal death or neonatal death

^c^Including previous caesarean section and/or uterine scar after gynecological surgery

^d^Prenatal care was considered inadequate if care did not begin before 14 completed weeks’ gestation. If care did begin before them, the percentage of prenatal visits was used to define four categories of prenatal care: inadequate (< 50% of the recommended number), intermediate (50–79%), adequate (80–109%), and adequate plus (≥ 110%). Then, women with missing ultrasounds were reclassified in the inadequate category (if the first trimester ultrasound or both of the latter ultrasounds were missing) or the intermediate category (if only the second or third ultrasound was missing)

^e^Small for gestational age: estimation of weight < 10th percentile and large for gestational age: estimation of weight or abdominal circumference > 95th percentile

^f^Defined as the occurrence of one or more of the following complications: gestational diabetes, pre-eclampsia, eclampsia, placenta abruption, HELLP syndrome, venous thrombosis, pulmonary embolism, severe sepsis, convulsions, diabetic ketoacidosis, coagulation disorder, cholestasis of pregnancy and complications due to uterine fibroids

^g^In case of multiple pregnancy, presentation of the first fetus

The overall cesarean rates significantly differed between Fr and SSA groups: 16.8% (370/2206) and 30.5% (458/1500) (*p* < 0.001). Whatever the type of cesarean considered, rates were systematically higher in the SSA than Fr group. Rates of cesarean before labor were 6.3% (139/2206) and 14.9% (224/1500), respectively, and rates during labor were 10.5% (231/2206) and 15.6% (234/1500). Rates of planned and emergency cesarean before labor and rates of cesarean during labor by onset of labor are in Additional file 3: Table S3.

Cesarean delivery profiles in Fr and SSA groups are presented in Table 2. The differences in cesarean rates between Fr and SSA groups were notable in some of the Robson categories: category 5 (previous uterine scar, single cephalic, ≥37 weeks), 6–7 (all breeches), 8 (all multiple pregnancy) and 10 (single cephalic, ≤ 36 weeks, including uterine scar). The category explaining most of the difference in cesarean rates between the Fr and SSA groups was category 5 (difference in absolute contribution: 9.3%) by both a higher cesarean rate and a greater size of the category in the SSA than Fr group. We further subdivided women in category 5 into two subcategories: a single uterine scar (category 5A) and multiple uterine scars (category 5B). As compared with category 5B, showing high cesarean rate in both Fr and SSA groups, for category 5A, the cesarean rates differ widely: 51.3 and 32.8% for SSA and Fr women respectively.

**Table 2:** Profiles of cesarean delivery according to maternal origin using the Robson classification

Robson category^a^	Number of CDs / number ofwomen in each category		Size of each category (%) (number of women in each category divided by the total number of women)			CD rate in each category (%) (number of cesarean deliveries in the category divided by the number of women in the category)			Contribution of each category (%) (number of cesarean deliveries in each category divided by the total number of women)		
	SSA group	Fr group	SSA group	Fr group	Difference (SSA-Fr)	SSA group	Fr group	Difference (SSA-Fr)	SSA group	Fr group	Difference (SSA-Fr)
1	26/193	64/737	12.9	33.5	−20.6	13.5	8.7	4.8	1.7	2.9	−1.2
2A	48/131	79/283	8.8	12.9	−4.1	36.6	27.9	8.7	3.2	3.6	−0.4
2B	7/7	16/16	0.5	0.7	−0.3	100.0	100.0	0.0	0.5	0.7	−0.3
1 + 2A + 2B	81/331	159/1036	22.2	47.1	−24.9	24.5	15.3	9.1	5.4	7.2	−1.8
3	27/486	7/526	32.6	23.9	8.7	5.6	1.3	4.2	1.8	0.3	1.5
4A	26/163	12/156	10.9	7.1	3.8	16	7.7	8.3	1.7	0.5	1.2
4B	17/17	9/9	1.1	0.4	0.7	100.0	100.0	0.0	1.1	0.4	0.7
3 + 4A + 4B	70/666	28/691	44.7	31.4	13.2	10.5	4.1	6.5	4.7	1.3	3.4
5A	99/193	41/125	13.0	5.7	7.3	51.3	32.8	18.5	6.6	1.9	4.8
5B	80/88	21/25	5.9	1.1	4.8	90.9	84.0	6.9	5.4	1.0	4.4
5A + 5B	181/284	63/152	19.0	6.9	12.1	63.7	41.4	22.3	12.1	2.9	9.3
6	7/8	41/61	0.5	2.8	−2.2	87.5	67.2	20.3	0.5	1.9	−1.4
7	29/33	17/34	2.2	1.5	0.7	87.9	50.0	37.9	1.9	0.8	1.2
8	36/60	26/78	4.0	3.5	0.5	60.0	33.3	26.7	2.4	1.2	1.2
9	10/11	2/2	0.7	0.1	0.6	90.9	100.0	−9.1	0.7	0.1	0.6
10	40/98	34/144	6.6	6.6	0.0	40.8	23.6	17.2	2.7	1.5	1.1
Total	458/1491^b^	370/2198^b^				30.7	16.8	13.9			

*Abbreviations*: *SSA group* women born in and originally from Sub-Saharan Africa, *Fr group* women born in and originally from mainland France, *CD* cesarean delivery

^a^Description of each category of Robson classification: 1 = Nulliparous, single cephalic, ≥37 weeks, spontaneous labor, 2 = Nulliparous, single cephalic, ≥37 weeks; 2A = induced - 2B = cesarean before labor, 3 = Multiparous (excluding previous uterine scar), single cephalic, ≥37 weeks, spontaneous labor, 4 = Multiparous (excluding previous uterine scar), single cephalic, ≥37 weeks; 4A = induced - 4B = cesarean before labor, 5A = One previous uterine scar, single cephalic, ≥37 weeks - 5B = More than one previous uterine scar, single cephalic, ≥37 weeks, 6 = All nulliparous breeches, 7 = All multiparous breeches (including previous uterine scar), 8 = All multiple pregnancies (including previous uterine scar), 9 = All abnormal lies (including previous uterine scar), 10 = All single cephalic, ≤ 36 weeks (including previous uterine scar)

^b^Missing data prevented the determination of the Robson category for 9 women of SSA group and 8 women of Fr Group

The association between maternal origin and cesarean delivery was then assessed within category 5A “single uterine scar, single, cephalic, ≥37 weeks”. Characteristics of women are in Additional file 1: Table S1 and Additional file 2: Table S2. Among the 318 women of this category, 69 had a cesarean delivery before labor, 71 had a cesarean during labor and 178 had a vaginal delivery. After multiple imputation and adjustment for medical factors, the association between maternal SSA origin and cesarean delivery before labor remained statistically significant (aOR = 2.30, 95% CI: 1.12–4.71), but when social deprivation was added to the model, the association was no longer significant (aOR = 1.45, 95% CI: 0.63–3.31) (Table 3). The risk of cesarean delivery during labor was higher for SSA than Fr women after adjustment for medical factors (aOR = 3.02, 95% CI: 1.47–6.23) and remained high after adjustment for both medical and social risk factors (aOR = 2.95, 95% CI: 1.35–6.44) (Table 4).

**Table 3:** Association between maternal origin and cesarean delivery before labor in the Robson 5A category (*n* = 318)

Variable	Cesarean delivery before labor versus trial of labor		
	OR [95% CI]	aOR^a^ [95% CI]	aOR^b^ [95% CI]
Group			
Fr	1	1	1
SSA	**1.95 [1.09–3.50]**	**2.30 [1.12–4.71]**	1.45 [0.63–3.31]
Maternal age (years) ^**c**^		1.06 [1.00–1.13]	1.07 [1.00–1.14]
Body mass index (kg/m^2^)^**c**^		1.04 [0.98–1.10]	1.03 [0.98–1.09]
Parity			
0–1		1	1
≥ 2		**0.24 [0.11–0.51]**	**0.23 [0.11–0.51]**
Medical risk level at the beginning of pregnancy^d^			
Low		1	1
High		1.49 [0.78–2.84]	1.55 [0.80–3.01]
Adequacy of prenatal care utilization^d^			
Inadequate		**3.76 [1.40–10.10]**	**3.68 [1.34–10.06]**
Intermediate		1.02 [0.27–3.89]	1.05 [0.27–4.16]
Adequate		1	1
Adequate plus		1.70 [0.60–4.80]	1.73 [0.60–4.99]
Estimation of fetal weight^d^			
Normal or small for gestational age		1	1
Large for gestational age		1.87 [0.40–8.72]	1.88 [0.40–8.86]
Complications during pregnancy^d^			
No		1	1
Yes		2.29 [0.99–5.31]	2.30 [0.98–5.43]
Social deprivation^d^			
No			1
Yes			**2.43 [1.14–5.18]**

*Abbreviations*: *OR* odds-ratio, *aOR* adjusted OR, *95% CI* 95% confidence interval, *Fr group* women born in mainland France and originally from mainland France, *SSA group* women born in Sub-Saharan Africa and originally from Sub-Saharan Africa. Statistically significant results appear in boldface

^a^Logistic regression models including all variables in the column (except social deprivation) + maternity unit of delivery; imputed data

^b^Logistic regression models including all variables in the column + maternity unit of delivery; imputed data

^**c**^Continuous variables

^d^See definitions in Table 1

**Table 4:** Association between maternal origin and cesarean delivery during labor in the 5A Robson category (*n* = 249)

Variable	Cesarean delivery during labor versus vaginal delivery		
	OR [95% CI]	aOR^a^ [95% CI]	aOR^b^ [95% CI]
Group			
Fr	1	1	1
SSA	**1.99 [1.11–3.56]**	**3.02 [1.47–6.23]**	**2.95 [1.35–6.44]**
Maternal age (years) ^**c**^		0.97 [0.91–1.03]	0.97 [0.91–1.03]
Body mass index (kg/m^2^)^**c**^		**1.08 [1.02–1.14]**	**1.08 [1.02–1.14]**
Parity			
0–1		1	1
≥ 2		**0.32 [0.16–0.67]**	**0.32 [0.16–0.67]**
Medical risk level at the beginning of pregnancy^d^			
Low		1	1
High		1.54 [0.80–2.99]	1.55 [0.80–3.00]
Adequacy of prenatal care utilization^d^			
Inadequate		1.65 [0.67–4.07]	1.65 [0.67–4.05]
Intermediate		1.07 [0.35–3.30]	1.08 [0.35–3.32]
Adequate		1	1
Adequate plus		2.14 [0.84–5.46]	2.14 [0.84–5.48]
Estimation of fetal weight^d^			
Normal or small for gestational age		1	1
Large for gestational age		**12.73 [1.71–94.47]**	**12.84 [1.73–95.33]**
Complications during pregnancy^d^			
No		1	1
Yes		0.98 [0.33–2.90]	0.98 [0.33–2.90]
Social deprivation^d^			
No			1
Yes			1.07 [0.51–2.25]

*Abbreviations*: *OR* odds-ratio, *aOR* adjusted OR, *95% CI* 95% confidence interval, *Fr group* women born in mainland France and originally from mainland France, *SSA group* women born in Sub-Saharan Africa and originally from Sub-Saharan Africa. Statistically significant results appear in boldface

^a^Logistic regression models including all variables in the column (except social deprivation) + maternity unit of delivery; imputed data

^b^Logistic regression models including all variables in the column + maternity unit of delivery; imputed data

^c^Continuous variables

^d^See definitions in Table 1

The results of the complete-cases analyses were similar to imputed results (Additional file 4: Table S4). The sensitivity analysis with maternal geographical origin defined only by the mother’s place of birth also provided similar results (Additional file 5: Table S5).

Women excluded because they were lost to follow-up, gave birth in a non-participating hospital or had missing data concerning the mode of delivery (*n* = 301) were younger, more often primiparous and socially deprived than those included in the analyses (*n* = 3706) (Additional file 6: Table S6).

## Discussion

The wide difference in cesarean rates between SSA and Fr groups was largely due to women who had one previous uterine scar. Among these women, the increased risk of cesarean delivery associated with SSA origin, whether performed before or during labor, was not explained by medical factors.

The main strengths of our study are its prospective and multicenter design and its large sample size. Help in completing questionnaires and their availability in four languages allowed for the participation of women with linguistic barriers or reading difficulties. The Robson classification provides an objective and clinically pertinent way to describe cesarean profiles. The richness of data collected in the cohort allowed for adjusting for main intermediate and confounding factors, both medical and socio-demographical [15]. The main limits of our study were the small size of women in the Robson 5A category, which limited statistical power, and the unavailability of information on complications during labor, such as fetal heart rate abnormalities and labor progression.

The increased rates of cesarean in SSA women, whatever the type of cesarean considered, were consistent with previous international literature [15, 29, 30]. Moreover, Minsart et al., using the Robson classification, also reported the important contribution of category 5 on differences in cesarean rates between native and SSA women in Belgium, which highlights the impact of the first cesarean delivery on subsequent deliveries for SSA women [30]. Further research is needed to identify modifiable risk factors of the first cesarean delivery and to understand the increased prevalence of women with uterine scar among SSA women.

To our knowledge, no previous study has assessed whether the differential use of cesarean delivery in SSA women was explained by medical factors. Indeed, in studies assessing the association between maternal origin and cesarean delivery, adjustment was performed simultaneously on medical and social factors, which did not allow for an interpretation of their respective roles. Thus, in the United Kingdom, Essex et al. found no significant association between mother’s ethnicity (“black” vs “white”) and cesarean delivery, whether elective or in an emergency, after adjustment for both medical and social factors [31]. Conversely, Von Katterfeld et al. found a significant association between mother’s country of birth (SSA vs Australia) and cesarean delivery, whether elective or in an emergency, after adjustment for both medical and social factors [32]. These heterogeneous results may be explained by methodological discrepancies (various definitions of maternal origin, classification of cesarean delivery and variables for adjustment), and national specificities of minority populations as well as barriers for access to care. No studies were performed specifically of the Robson 5A category.

Our results suggesting that medical factors do not explain the increased risk of cesarean delivery before labor among SSA women in the Robson 5A category highlight the fact that social deprivation play an important role in this association. Social deprivation in the group of women originated from SSA may be a marker of more recent migration, which may affect the decision for cesarean delivery before labor because of a possible apprehension from physicians to perform a trial of labor in cases of uterine scar when the surgery was performed in Africa. The role of social deprivation might also reflect medically unjustified differential care according to socioeconomic status, thereby demonstrating the poor capacity of women in the least favorable social positions to negotiate care. Low health literacy that may be due to a disadvantaged social context or to a recent migration may hinders the chance of women’s participation in the decisions that concern her. One limitation of this study was that we had no data to assess literacy in women’s health.

For cesarean delivery during labor, the persistence of an increased risk among SSA women after adjustment for both medical and socioeconomic factors could be explained by a different pattern of labor between native French and SSA women. In fact, several authors described specific features of the pelvic anatomy in women of Black African origin [33, 34]. These features could lead to more complications during labor or may need appropriate management, as suggested by older studies [35]. These older notions, inherited from physical anthropology and scientifically questionable methods, may no longer be valid today with the later age of puberty and women’s less exposure to heavy loads and less likely exposure to childhood poliomyelitis. Further research is needed to assess the impact of maternal ethnicity on pelvic morphology and the progress of labor.

A more robust hypothesis is that the difference in cesarean rates could be explained by communication factors. A linguistic barrier preventing good communication on symptoms, advice and explanations could more frequently lead to cesarean delivery during labor [36–39]. The management of pain might differ by maternal origin and may influence the mode of delivery. Finally, the existence of implicit bias (unconscious negative feelings or stereotypes against a group) has been highlighted among healthcare professionals [40–42]. This bias can lead to medically unjustified differential care, particularly in uncertain or stressful situations such as delivery [40, 43, 44]. Improving intercultural communication skills among health care providers and raising awareness of implicit bias may help reduce the gap between the cesarean rates of French and SSA groups by preventing non-medically justified cesarean deliveries. Studies using mixed methods are needed to test these hypotheses.

## Conclusions

We showed a wide difference in overall caesarean delivery rates between women from France and Sub-Saharan Africa and that the presence of one previous uterine scar (Robson 5A category) explained most of this difference. Within this group, medical factors alone do not explain the increased risk of cesarean delivery before or during labor in Sub-Saharan Africa women. In a context of wide ethnic disparities, our results — highlighting the importance of non-medical factors in differential rates of cesarean by maternal geographical origin — are important in understanding the root causes of these disparities.

## Additional files

Additional file 1: **Table S1.** Cesarean before labor vs Trial of labor - Characteristics of women. (DOCX 19 kb)

Additional file 2: **Table S2.** Cesarean during labor vs vaginal delivery - Characteristics of women. (DOCX 19 kb)

Additional file 3: **Table S3.** Differential rates of cesarean between Fr and SSA women. (DOCX 14 kb)

Additional file 4: **Table S4.** Association between maternal origin and cesarean delivery within the Robson 5A category: complete cases analysis. (DOCX 17 kb)

Additional file 5: **Table S5.** Association between mother’s place of birth and cesarean delivery within the Robson 5A category. (DOCX 17 kb)

Additional file 6: **Table S6** Comparisons of women included and excluded of the analysis. (DOCX 18 kb)

## Data Availability

The data underlying the findings cannot be made freely available because of ethical and legal restrictions (French laws on data protection). Indeed, the present analysis involves a large number of variables that, combined, could be used to re-identify the participating women or children based on a few key characteristics, and then to have access to other personal data. Therefore, the French ethical authority (Commission Nationale de l’Informatique et des Libertés) strictly forbids making such data freely available. However, all relevant data can be obtained upon request from the PreCARE steering committee. Readers may contact Professor Elie AZRIA (eazria@hpsj.fr) to request the data.

## Abbreviations

aOR: Adjusted odds ratio
SSA: Sub-Saharan Africa
