# Supplementary material for: Differential rates of cesarean delivery by maternal geographical origin: a cohort study in France
Source: BMC Pregnancy Childbirth. 2019 Jun 27;19:217. doi: 10.1186/s12884-019-2364-x (PMC6598349; doi:10.1186/s12884-019-2364-x)
Supplement: Supplementary file 1 — Table S1. Cesarean before labor vs Trial of labor - Characteristics of women. (DOCX 19 kb) [file 12884_2019_2364_MOESM1_ESM.docx]

**Additional file 1: Table S1: Cesarean before labor vs Trial of labor - Characteristics of women**

| Characteristic |  | Trial of labor n=249 | | Cesarean before labor n=69 | | Missing data | |
| --- | --- | --- | --- | --- | --- | --- | --- |
|  |  | N | % | N | % | N | % |
| Group | Fr | 106 | 42.6 | 19 | 27.5 | 0 | 0.0 |
|  | SSA | 143 | 57.4 | 50 | 72.5 |  |  |
| Age (years) | < 25 | 17 | 6.8 | 3 | 4.3 | 0 | 0.0 |
|  | 25-29 | 60 | 24.1 | 15 | 21.7 |  |  |
|  | 30-34 | 90 | 36.1 | 23 | 33.3 |  |  |
|  | ≥ 35 | 82 | 32.9 | 28 | 40.6 |  |  |
| Body mass index (kg/m²) | <24.9 | 116 | 50.4 | 22 | 33.3 | 22 | 6.9 |
|  | 25-29.9 | 67 | 29.1 | 26 | 39.4 |  |  |
|  | ≥30 | 47 | 20.4 | 18 | 27.3 |  |  |
| Parity | 0-1* | 151 | 60.6 | 52 | 75.4 | 0 | 0.0 |
|  | ≥2 | 98 | 39.4 | 17 | 24.6 |  |  |
| Medical risk level at the beginning of pregnancy^‡^ | Low | 174 | 70.2 | 43 | 62.3 | 1 | 0.3 |
|  | High | 74 | 29.8 | 26 | 37.7 |  |  |
| Education | ≤ Primary school | 29 | 11.7 | 14 | 20.6 | 2 | 0.6 |
|  | Middle school | 50 | 20.2 | 14 | 20.6 |  |  |
|  | High school | 52 | 21.0 | 14 | 20.6 |  |  |
|  | University | 117 | 47.2 | 26 | 38.2 |  |  |
| Social deprivation^‡^ | No | 162 | 65.1 | 27 | 39.1 | 0 | 0.0 |
|  | Yes | 87 | 34.9 | 42 | 60.9 |  |  |
| Adequacy of prenatal care utilization^‡^ | Inadequate | 88 | 36.7 | 37 | 55.2 | 11 | 3.5 |
|  | Intermediate | 40 | 16.7 | 5 | 7.5 |  |  |
|  | Adequate | 47 | 19.6 | 7 | 10.4 |  |  |
|  | Adequate plus | 65 | 27.1 | 18 | 26.9 |  |  |
| Estimation of fetal weight^‡^ | Small for gestational age or Normal | 230 | 97.5 | 62 | 93.9 | 16 | 5.0 |
|  | Large for gestational age | 6 | 2.5 | 4 | 6.1 |  |  |
| Complications of pregnancy^‡^ | No | 225 | 90.4 | 54 | 78.3 | 0 | 0.0 |
|  | Yes | 24 | 9.6 | 15 | 21.7 |  |  |
| Abbreviations : Fr = women born in mainland France and originally from mainland France, SSA = women born in Sub-Saharan Africa and originally from Sub-Saharan Africa. | | | | | | | |
| * Women with parity=0 are women with uterine scar after gynecological surgery | | | | | | | |
| ^‡^ See definitions in Table 1 |  |  |  |  |  |  |  |
