# Supplementary material for: Differential rates of cesarean delivery by maternal geographical origin: a cohort study in France
Source: BMC Pregnancy Childbirth. 2019 Jun 27;19:217. doi: 10.1186/s12884-019-2364-x (PMC6598349; doi:10.1186/s12884-019-2364-x)
Supplement: Supplementary file 2 — Table S2. Cesarean during labor vs vaginal delivery - Characteristics of women. (DOCX 19 kb) [file 12884_2019_2364_MOESM2_ESM.docx]

**Additional file 2: Table S2: Cesarean during labor vs vaginal delivery - Characteristics of women**

| Characteristic |  | Vaginal delivery n=178 | | Cesarean during labor n=71 | | Missing data | |
| --- | --- | --- | --- | --- | --- | --- | --- |
|  |  | N | % | N | % | N | % |
| Group | Fr | 84 | 47.2 | 22 | 31.0 | 0 | 0.0 |
|  | SSA | 94 | 52.8 | 49 | 69.0 |  |  |
| Age (years) | < 25 | 10 | 5.6 | 7 | 9.9 | 0 | 0.0 |
|  | 25-29 | 41 | 23.0 | 19 | 26.8 |  |  |
|  | 30-34 | 61 | 34.3 | 29 | 40.8 |  |  |
|  | ≥ 35 | 66 | 37.1 | 16 | 22.5 |  |  |
| Body mass index (kg/m²) | <24.9 | 86 | 52.8 | 30 | 44.8 | 19 | 7.6 |
|  | 25-29.9 | 49 | 30.1 | 18 | 26.9 |  |  |
|  | ≥30 | 28 | 17.2 | 19 | 28.4 |  |  |
| Parity | 0-1* | 99 | 55.6 | 52 | 73.2 | 0 | 0.0 |
|  | ≥2 | 79 | 44.4 | 19 | 26.8 |  |  |
| Medical risk level at the beginning of pregnancy^‡^ | Low | 128 | 72.3 | 46 | 64.8 | 1 | 0.4 |
|  | High | 49 | 27.7 | 25 | 35.2 |  |  |
| Education | ≤ Primary school | 19 | 10.7 | 10 | 14.1 | 1 | 0.4 |
|  | Middle school | 35 | 19.8 | 15 | 21.1 |  |  |
|  | High school | 32 | 18.1 | 20 | 28.2 |  |  |
|  | University | 91 | 51.4 | 26 | 36.6 |  |  |
| Social deprivation^‡^ | No | 120 | 67.4 | 42 | 59.2 | 0 | 0.0 |
|  | Yes | 58 | 32.6 | 29 | 40.8 |  |  |
| Adequacy of prenatal care utilization^‡^ | Inadequate | 61 | 35.1 | 27 | 40.9 | 9 | 3.6 |
|  | Intermediate | 32 | 18.4 | 8 | 12.1 |  |  |
|  | Adequate | 36 | 20.7 | 11 | 16.7 |  |  |
|  | Adequate plus | 45 | 25.9 | 20 | 30.3 |  |  |
| Estimation of fetal weight^‡^ | Small for gestational age or Normal | 166 | 98.8 | 64 | 94.1 | 13 | 5.2 |
|  | Large for gestational age | 2 | 1.2 | 4 | 5.9 |  |  |
| Complications of pregnancy^‡^ | No | 161 | 90.4 | 64 | 90.1 | 0 | 0.0 |
|  | Yes | 17 | 9.6 | 7 | 9.9 |  |  |
| Abbreviations: Fr = women born in mainland France and originally from mainland France, SSA = women born in Sub-Saharan Africa and originally from Sub-Saharan Africa. | | | | | | | |
| * Women with parity=0 are women with uterine scar after gynecological surgery | | | | | | | |
| ^‡^ See definitions in Table 1 |  |  |  |  |  |  |  |
