# Supplementary material for: Differential rates of cesarean delivery by maternal geographical origin: a cohort study in France
Source: BMC Pregnancy Childbirth. 2019 Jun 27;19:217. doi: 10.1186/s12884-019-2364-x (PMC6598349; doi:10.1186/s12884-019-2364-x)
Supplement: Supplementary file 3 — Table S3. Differential rates of cesarean between Fr and SSA women. (DOCX 14 kb) [file 12884_2019_2364_MOESM3_ESM.docx]

**Additional file 3: Table S3: Differential rates of cesarean between Fr and SSA women**

| Rates of cesarean | Fr Group | | SSA Group | |
| --- | --- | --- | --- | --- |
|  | n= | 2206 | n= | 1500 |
|  | N | % | N | % |
| Cesarean delivery (overall) | 370 | (16.8) | 458 | (30.5) |
| Cesarean before labor | 139 | (6.3) | 224 | (14.9) |
| Planned | 75 | (3.4) | 136 | (9.1) |
| Emergency | 55 | (2.5) | 74 | (4.9) |
| Missing data | 9 | (0.4) | 14 | (0.9) |
| Cesarean during labor | 231 | (10.5) | 234 | (15.6) |
| Spontaneous labor | 117 | (5.3) | 120 | (8.0) |
| Induced labor | 114 | (5.2) | 114 | (7.6) |
| Abbreviations: Fr group = women born in mainland France and originally from mainland France, SSA group = women born in Sub-Saharan Africa and originally from Sub-Saharan Africa | | | | |
