# Supplementary material for: Differential rates of cesarean delivery by maternal geographical origin: a cohort study in France
Source: BMC Pregnancy Childbirth. 2019 Jun 27;19:217. doi: 10.1186/s12884-019-2364-x (PMC6598349; doi:10.1186/s12884-019-2364-x)
Supplement: Supplementary file 4 — Table S4. Association between maternal origin and cesarean delivery within the Robson 5A category: complete cases analysis. (DOCX 17 kb) [file 12884_2019_2364_MOESM4_ESM.docx]

**Additional file 4: Table S4: Association between maternal origin and cesarean delivery within the Robson 5A category: complete cases analysis**

| Variable |  | Cesarean delivery before labor versus trial of labor | | Cesarean delivery during labor versus vaginal delivery | |
| --- | --- | --- | --- | --- | --- |
|  |  | aOR* [95% CI] | aOR** [95% CI] | aOR* [95% CI] | aOR** [95% CI] |
| Group | Fr | 1 | 1 | 1 | 1 |
|  | SSA | **2.40 [1.14-5.08]** | 1.28 [0.53-3.08] | **2.96 [1.35-6.47]** | **3.03 [1.30-7.08]** |
| Maternal age (years) **^†^** |  | 1.05 [0.98-1.12] | 1.06 [0.99-1.14] | 0.94 [0.88-1.01] | 0.94 [0.88-1.01] |
| Body mass index (kg/m²)**^†^** |  | 1.04 [0.98-1.10] | 1.03 [0.97-1.09] | **1.09 [1.02-1.16]** | **1.09 [1.02-1.16]** |
| Parity | 0-1 | 1 | 1 | 1 | 1 |
|  | ≥ 2 | **0.20 [0.09-0.47]** | **0.19 [0.08-0.45]** | **0.37 [0.17-0.81]** | **0.37 [0.17-0.82]** |
| Medical risk level at the beginning of pregnancy^‡^ | Low | 1 | 1 | 1 | 1 |
|  | High | 1.55 [0.78-3.10] | 1.65 [0.81-3.37] | 1.86 [0.91-3.81] | 1.86 [0.91-3.80] |
| Adequacy of prenatal care utilization^‡^ | Inadequate | **3.45 [1.25 -9.52]** | **3.48 [1.23-9.88]** | 1.92 [0.72-5.14] | 1.92 [0.72-5.15] |
|  | Intermediate | 0.98 [0.23-4.17] | 1.10 [0.25-4.86] | 1.09 [0.32-3.71] | 1.08 [0.32-3.70] |
|  | Adequate | 1 | 1 | 1 | 1 |
|  | Adequate plus | 1.66 [0.57-4.81] | 1.73 [0.57-5.18] | 2.05 [0.74-5.73] | 2.05 [0.73-5.72] |
| Estimation of fetal weight^‡^ | Normal or small for gestational age | 1 | 1 | 1 | 1 |
|  | Large for gestational age | 1.35 [0.27-6.81] | 1.40 [0.28-7.12] | **13.56 [1.65-111.26]** | **13.46 [1.63-110.86]** |
| Complications during pregnancy^‡^ | No | 1 | 1 | 1 | 1 |
|  | Yes | 2.13 [0.87-5.21] | 2.16 [0.86-5.43] | 0.87 [0.27-2.81] | 0.87 [0.27-2.82] |
| Social deprivation^‡^ | No |  | 1 |  | 1 |
|  | Yes |  | **3.27 [1.43 -7.48]** |  | 0.94 [0.42-2.11] |
| **Abbreviations**: aOR (adjusted OR), 95% CI (95% confidence interval), Fr group = women born in mainland France and originally from mainland France, SSA group = women born in Sub-Saharan Africa and originally from Sub-Saharan Africa. Statistically significant results appear in boldface. | | | | |  |
| *Logistic regression models including all variables in the column + maternity unit of delivery  ** Logistic regression models including all variables in the column + maternity unit of delivery + social deprivation | | | | |  |
| **^†^**Continuous variables | | | | |  |
| ^‡^See definitions in Table 1 | | | | |  |
