# Supplementary material for: Differential rates of cesarean delivery by maternal geographical origin: a cohort study in France
Source: BMC Pregnancy Childbirth. 2019 Jun 27;19:217. doi: 10.1186/s12884-019-2364-x (PMC6598349; doi:10.1186/s12884-019-2364-x)
Supplement: Supplementary file 5 — Table S5. Association between mother’s place of birth and cesarean delivery within the Robson 5A category. (DOCX 17 kb) [file 12884_2019_2364_MOESM5_ESM.docx]

**Additional file 5: Table S5: Association between mother’s place of birth and cesarean delivery within the Robson 5A category**

| Variable |  | Cesarean delivery before labor versus trial of labor | | Cesarean delivery during labor versus vaginal delivery | |
| --- | --- | --- | --- | --- | --- |
|  |  | aOR* [95% CI] | aOR** [95% CI] | aOR* [95% CI] | aOR** [95% CI] |
| Place of birth | France | 1 | 1 | 1 | 1 |
|  | Sub-Saharan Africa | **2.04 [1.18-3.53]** | 1.28 [0.53-3.08] | **2.26 [1.27-4.01]** | **2.25 [1.23-4.11]** |
| Maternal age (years) **^†^** |  | 1.05 [0.99-1.11] | 1.06 [0.99-1.14] | 0.96 [0.90-1.02] | 0.96 [0.90-1.02] |
| Body mass index (kg/m²)**^†^** |  | 1.03 [0.98-1.08] | 1.03 [0.97-1.09] | **1.07 [1.02-1.12]** | **1.07 [1.02-1.12]** |
| Parity | 0-1 | 1 | 1 | 1 | 1 |
|  | ≥ 2 | **0.22 [0.11-0.43]** | **0.19 [0.08-0.45]** | **0.32 [0.17-0.60]** | **0.32 [0.17-0.60]** |
| Medical risk level at the beginning of pregnancy^‡^ | Low | 1 | 1 | 1 | 1 |
|  | High | **1.75 [1.01-3.02]** | 1.65 [0.81-3.37] | 1.36 [0.77-2.42] | 1.36 [0.77-2.42] |
| Adequacy of prenatal care utilization^‡^ | Inadequate | **3.31 [1.44 -7.61]** | **3.48 [1.23-9.88]** | 1.06 [0.51-2.21] | 1.06 [0.51-2.21] |
|  | Intermediate | 2.01 [0.73-5.58] | 1.10 [0.25-4.86] | 0.44 [0.16-1.23] | 0.44 [0.16-1.24] |
|  | Adequate | 1 | 1 | 1 | 1 |
|  | Adequate plus | 1.72 [0.73-4.06] | 1.73 [0.57-5.18] | 1.37 [0.65-2.89] | 1.37 [0.65-2.89] |
| Estimation of fetal weight^‡^ | Normal or small for gestational age | 1 | 1 | 1 | 1 |
|  | Large for gestational age | 3.00 [0.91-9.83] | 1.40 [0.28-7.12] | **5.04 [1.07-23.80]** | **5.06 [1.07-23.89]** |
| Complications during pregnancy^‡^ | No | 1 | 1 | 1 | 1 |
|  | Yes | 1.71 [0.81-3.62] | 2.16 [0.86-5.43] | 0.58 [0.22-1.54] | 0.58 [0.22-1.54] |
| Social deprivation^‡^ | No |  | 1 |  | 1 |
|  | Yes |  | **3.27 [1.43 -7.48]** |  | 1.01 [0.55-1.87] |
| Abbreviations: aOR (adjusted OR), 95% CI (95% confidence interval), statistically significant results appear in boldface | | | | |  |
| *Logistic regression models including all variables in the column + maternity unit of delivery  ** Logistic regression models including all variables in the column + maternity unit of delivery + social deprivation | | | | |  |
| **^†^**Continuous variables | | | | |  |
| ^‡^ See definitions in Table 1 | | | | |  |
