# Supplementary material for: Differential rates of cesarean delivery by maternal geographical origin: a cohort study in France
Source: BMC Pregnancy Childbirth. 2019 Jun 27;19:217. doi: 10.1186/s12884-019-2364-x (PMC6598349; doi:10.1186/s12884-019-2364-x)
Supplement: Supplementary file 6 — Table S6 Comparisons of women included and excluded of the analysis. (DOCX 18 kb) [file 12884_2019_2364_MOESM6_ESM.docx]

**Additional file 6: Table S6: Comparisons of women included and excluded of the analysis**

| Characteristic |  | Women included | | Women excluded* | | p |
| --- | --- | --- | --- | --- | --- | --- |
|  |  | n=  n | 3706  (%) | n=  n | 301  (%) |  |
| Age (years) | < 25 | 418 | (11.3) | 58 | (19.3) | <0.01 |
|  | 25-29 | 1069 | (28.8) | 84 | (27.9) |  |
|  | 30-34 | 1346 | (36.3) | 79 | (26.2) |  |
|  | ≥ 35 | 873 | (23.6) | 80 | (26.6) |  |
| Body mass index (kg/m²) | <24,9 | 2346 | (67.2) | 169 | (71.6) | 0.38 |
|  | 25-29,9 | 682 | (19.5) | 40 | (16.9) |  |
|  | ≥30 | 462 | (13.2) | 27 | (11.4) |  |
| Parity | 0 | 1651 | (44.6) | 154 | (57.5) | <0.01 |
|  | 1 | 1098 | (29.6) | 65 | (24.2) |  |
|  | ≥2 | 955 | (25.8) | 49 | (18.3) |  |
| Medical risk level at the beginning of pregnancy^†^ | Low | 2912 | (78.8) | 203 | (81.2) | 0.37 |
|  | High | 783 | (21.2) | 47 | (18.8) |  |
| Previous uterine scar^†^ | No | 3188 | (86.1) | 243 | (90.7) | 0.11 |
|  | One uterine scar | 377 | (10.2) | 18 | (6.7) |  |
|  | More than one uterine scar | 138 | (3.7) | 7 | (2.6) |  |
| Education | ≤ Primary school | 334 | (9.1) | 24 | (8.0) | 0.86 |
|  | Middle school | 591 | (16.1) | 52 | (17.3) |  |
|  | High school | 664 | (18.0) | 52 | (17.3) |  |
|  | University | 2092 | (56.8) | 173 | (57.5) |  |
| Social deprivation^†^ | No | 2567 | (69.3) | 178 | (59.1) | <0.01 |
|  | Yes | 1136 | (30.7) | 123 | (40.9) |  |
| Multiple pregnancy | No | 3569 | (96.3) | 265 | (94.3) | 0.09 |
|  | Yes | 137 | (3.7) | 16 | (5.7) |  |
| * Women from the Fr or SSA group and excluded because they gave birth in a non-participating hospital, were lost to follow-up or had missing data concerning their mode of delivery  **^†^**See definitions in Table 1 | | | | | | |
